# Phytochemical Modulators of Nociception: A Review of Cannabis Terpenes in Chronic Pain Syndromes

**DOI:** 10.3390/ph18081100

**Published:** 2025-07-24

**Authors:** Aniello Alfieri, Sveva Di Franco, Vincenzo Maffei, Pasquale Sansone, Maria Caterina Pace, Maria Beatrice Passavanti, Marco Fiore

**Affiliations:** 1 Department of Women, Child and General and Specialized Surgery, University of Campania “Luigi Vanvitelli”, 80138 Naples, Italy; aniello.alfieri@unicampania.it (A.A.); sveva.difranco@unicampania.it (S.D.F.); pasquale.sansone@unicampania.it (P.S.); mariacaterina.pace@unicampania.it (M.C.P.); mariabeatrice.passavanti@unicampania.it (M.B.P.); 2 Department of Anaesthesia and Intensive Care, A.O.R.N. Antonio Cardarelli, 80131 Naples, Italy; vincenzo.maffei@aocardarelli.it; 3 Department of Anaesthesia and Intensive Care, P.O. Pellegrini, 80134 Naples, Italy

**Keywords:** medical cannabis, cannabis terpenes, chronic pain modulation, phytotherapeutics, analgesic mechanism, neuroinflammation

## Abstract

*Cannabis sativa* L. is a phytochemically rich plant with therapeutic potential across various clinical domains, including pain, inflammation, and neurological disorders. Among its constituents, terpenes are gaining recognition for their capacity to modulate the pathophysiological processes underlying chronic pain syndromes. Traditionally valued for their aromatic qualities, terpenes such as myrcene, β-caryophyllene (BCP), limonene, pinene, linalool, and humulene have demonstrated a broad spectrum of biological activities. Beyond their observable analgesic, anti-inflammatory, and anxiolytic outcomes, these compounds exert their actions through distinct molecular mechanisms. These include the activation of cannabinoid receptor type 2 (CB_2_), the modulation of transient receptor potential (TRP) and adenosine receptors, and the inhibition of pro-inflammatory signalling pathways such as Nuclear Factor kappa-light-chain-enhancer of activated B cells (NF-κB) and Cyclooxygenase-2 (COX-2). This narrative review synthesizes the current preclinical and emerging clinical data on terpene-mediated analgesia, highlighting both monoterpenes and sesquiterpenes, and discusses their potential for synergistic interaction with cannabinoids, the so-called entourage effect. Although preclinical findings are promising, clinical translation is limited by methodological variability, the lack of standardized formulations, and insufficient pharmacokinetic characterization. Further human studies are essential to clarify their therapeutic potential.

## 1. Introduction

Chronic pain is a complex condition characterized by a dynamic interplay among the neuronal, immune, and vascular systems [1,2]. Affecting a substantial portion of the global population, its prevalence is increasing in parallel with global ageing, posing significant therapeutic challenges and adversely impacting quality of life [3]. Beyond peripheral and central sensitization, chronic pain is sustained by maladaptive synaptic remodelling, including long-term potentiation in the spinal dorsal horn [4], the structural reorganization of limbic circuits [5], and prolonged glial neuroinflammation mediated by Interleukin 1 beta (IL-1β), Tumour Necrosis Factor alpha (TNF-α), and chemokines that amplify nociceptive signalling [6].

Modern perspectives on chronic pain emphasize profound plastic reorganization at both the functional and structural levels within the peripheral and central nervous systems, particularly involving limbic structures such as the anterior cingulate cortex (ACC), amygdala, and bed nucleus of the stria terminalis (BNST), which are closely associated with the affective dimension of pain [7]. This maladaptive plasticity underpins the shift from acute nociception—a protective physiological mechanism—to chronic pain, a pathological condition [1]. Central sensitization, a form of synaptic plasticity, drives this transition by enhancing the excitability and response of nociceptive pathways in the spinal cord and supraspinal structures [8]. Clinically, this manifests as allodynia and hyperalgesia, symptoms closely linked to neuroinflammation [8,9].

Neuroinflammation is orchestrated by the glial cells—primarily microglia and astrocytes—which, upon activation by persistent nociceptive input or injury, release pro-inflammatory mediators, neurotransmitters, and neuromodulators. Microglia act as innate immune sentinels, initiating and amplifying the inflammatory response, whereas astrocytes modulate synaptic homeostasis and sustain the inflammatory milieu over time [10]. These molecules influence synaptic transmission and perpetuate the chronic pain state [9,11]. Notably, the emotional and cognitive dimensions of chronic pain, such as anxiety and depression, are associated with maladaptive plasticity in areas like the ACC, amygdala, and BNST [9,12]. Mitochondrial dysfunction, oxidative stress, and epigenetic alterations in nociceptors further sensitize pain pathways, while microvascular changes facilitate immune cell infiltration [8].

Given the multifactorial nature of chronic pain—including synaptic plasticity, neuro-immune interactions, and glial activation—single-target pharmacological approaches often fail to provide adequate and sustained relief. Conventional pharmacological strategies for chronic pain vary substantially depending on the underlying pathophysiological mechanism—whether nociceptive, neuropathic, inflammatory, or mixed in nature. For instance, nociceptive pain typically responds to nonsteroidal anti-inflammatory drugs (NSAIDs) whereas neuropathic pain requires neuromodulatory agents like gabapentin, pregabalin, tricyclic antidepressants (e.g., amitriptyline), or serotonin–norepinephrine reuptake inhibitors (e.g., duloxetine), which are considered first-line treatments for many chronic pain syndromes [13,14]. However, these agents are frequently associated with dose-limiting adverse effects. Gabapentin and pregabalin can cause sedation, dizziness, peripheral edema, and cognitive impairment, particularly in older adults [15]; amitriptyline is often limited by anticholinergic side effects such as dry mouth, urinary retention, and orthostatic hypotension [16]; while duloxetine may lead to nausea, insomnia, increased blood pressure, and withdrawal symptoms upon discontinuation [17].

NSAIDs—particularly ibuprofen, naproxen, and diclofenac—may be useful in inflammatory conditions such as osteoarthritis but are limited by gastrointestinal and cardiovascular risks when used long-term [18,19]. Opioids have long represented a cornerstone in the management of moderate to severe acute and chronic pain, due to their potent analgesic properties mediated primarily through μ-opioid receptor agonism. Despite their efficacy, the widespread use of opioids is increasingly challenged by a range of serious adverse effects, including tolerance, dependence, opioid-induced hyperalgesia, constipation, and the risk of overdose and misuse [20]. For this reason, opioids are generally reserved for selected cases of refractory pain, given their potential for dependence, tolerance, and opioid-induced hyperalgesia, and are no longer recommended as a first-line therapy in most chronic pain guidelines [21].

In this context, phytochemicals—bioactive plant-derived compounds—are emerging as promising modulators of pain signalling [22]. Belonging to diverse chemical classes such as flavonoids, terpenoids, and alkaloids, these compounds, though non-essential for plant growth, play key ecological roles and exhibit extensive pharmacological activity [23].

Preclinical studies have demonstrated that a variety of phytochemicals can modulate neuronal excitability by interacting with the key ion channels involved in nociceptive transmission, including voltage-gated sodium channels (Nav), voltage-gated calcium channels (Cav), voltage-gated potassium channels (Kv), and the transient receptor potential vanilloid 1 channel (TRPV1) [24,25]. In addition to modulating ion flux, several compounds inhibit inflammatory pathways by targeting enzymes such as COX-2, modulating glial activation, and regulating transcription factors NF-κB [26]. Phytochemicals such as curcumin, resveratrol, and capsaicin have shown analgesic potential through the suppression of pro-inflammatory cytokines, reduction in oxidative stress, and direct receptor-mediated mechanisms [22].

Among phytochemicals, those derived from *Cannabis sativa* are of growing relevance to pain management. The plant contains over 500 compounds, notably cannabinoids (e.g., Δ9-Tetrahydrocannabinol, THC, Cannabidiol, and CBD) and a wide range of terpenes [27,28]. Cannabinoids, especially via the cannabinoid receptor type 1 (CB_1_) and CB_2_ receptors, exert analgesic actions, but THC-related side effects limit their use [29]. Terpenes, responsible for the plant’s aroma, are increasingly recognized for their pharmacological activity and their ability to enhance cannabinoid-mediated responses through the so-called “entourage effect” [30,31].

Notably, terpenes may also act independently of cannabinoids, targeting distinct molecular pathways involved in nociception and neuroinflammation. Preclinical studies suggest that several terpenes possess pleiotropic mechanisms of action, potentially contributing autonomously or synergistically to analgesia [28]. However, further validation in human models remains necessary.

This narrative review critically examines the scientific literature on the role of *Cannabis sativa* terpenes in nociceptive modulation, with an emphasis on chronic pain syndromes. Its aims are to identify the most prevalent terpenes and their pharmacodynamics; evaluate preclinical efficacy in chronic pain models; explore molecular mechanisms including cannabinoid-independent pathways; assess therapeutic profiles, safety, and pharmacokinetics; highlight research gaps; and propose directions for future investigation.

### Methods

An extensive review of the literature was conducted to examine the analgesic effects of terpenes found in *Cannabis sativa*, with a particular focus on their applications in chronic pain. The review encompassed both preclinical studies (including in vitro and in vivo models) and clinical investigations in humans (randomized controlled trials, RCTs, observational studies, case reports), as well as relevant review articles, in order to provide a comprehensive and up-to-date overview of the topic.

Bibliographic research was carried out by consulting major scientific databases (PubMed/MEDLINE, Embase, and Web of Science), using combinations of keywords such as “terpenes,” “cannabis,” and “chronic pain,” combined using Boolean operators. Particular attention was given to studies published in the last 15 years (2010–2025), although seminal earlier works—particularly those frequently cited or deemed relevant, such as studies from the 1990s–2000s on the properties of myrcene or menthol—were also included. The search was updated through June 2025 to capture the most recent evidence, including potentially relevant studies published in 2024. Additionally, reference lists of identified papers were examined using a snowballing approach to retrieve further pertinent sources. No language restrictions were applied: relevant non-English studies (e.g., in Italian, Spanish, Portuguese, or German) with English abstracts were also included.

This review included a broad range of studies that collectively illuminate the analgesic potential of cannabis-derived terpenes. Preclinical investigations encompassed in vitro experiments aimed at identifying molecular targets and elucidating the mechanisms of action of isolated terpenes. In parallel, in vivo studies employing animal models of pain—both acute and chronic—were considered when they assessed the antinociceptive or analgesic properties of individual terpenes or terpene-rich essential oils. These studies involved diverse models of inflammatory, neuropathic, and visceral pain and typically evaluated outcomes such as pain thresholds, behavioural responses (including hot plate, formalin, and writhing tests, or assessments of mechanical allodynia), as well as the presence of edema and alterations in inflammatory biomarkers.

Clinical evidence was also included, focusing on controlled trials—whether randomized or crossover in design—that examined the effectiveness of terpenes or terpene-based preparations in treating chronic pain of various origins. These trials addressed a spectrum of conditions such as cancer-related pain, arthritis, migraine, diabetic neuropathy, fibromyalgia, and myofascial pain syndrome, often in the context of aromatherapy or topical applications. Complementary data from observational studies and case series enriched the clinical perspective.

In addition to primary research, the review incorporated systematic and narrative reviews as well as meta-analyses that directly addressed the role of terpenes in pain modulation, whether within the broader context of medical cannabis or in studies focused specifically on the therapeutic properties of essential oils and their active constituents. Furthermore, phytochemical and pharmacokinetic studies were examined to contextualize the pharmacognostic framework. These included analyses of terpene profiles in different *Cannabis sativa* chemovars, along with investigations into the absorption, distribution, metabolism, and excretion of terpenes in both animal and human models.

## 2. Phytochemical Modulation of Nociception: General Mechanisms

### 2.1. Molecular Targets and Mechanisms of Action

The use of phytochemicals as modulators of pain sensitivity rests on their ability to interfere with the complex network of molecular components involved in nociceptive transmission and neuroinflammation. These plant-derived compounds act concertedly on voltage-gated ion channels, TRP receptors, regulatory pathways of pro-inflammatory transcription factors, and, not least, targets within the endocannabinoid system, outlining a multi-target profile that transcends the conventional pharmacological approach [32].

Preclinical evidence in recent years has confirmed that flavonoids such as quercetin and myricetin directly inhibit Nav1.7 and Nav1.8 sodium channels and L-type Cav channels. Quercetin binds with high affinity to the voltage sensor domains of Nav channels, dose-dependently reducing sodium currents while simultaneously suppressing COX-2 expression, thereby lowering prostaglandin synthesis and attenuating peripheral sensitization [24]. Simultaneously, myricetin inhibits p38 mitogen-activated protein kinase (MAPK) phosphorylation and Protein Kinase C (PKC)-dependent signalling, limiting glutamate release in the spinal dorsal horn and normalizing aberrant neuronal excitability levels, as demonstrated in murine models of peripheral neuropathy [33].

The modulation of TRP receptors constitutes another mechanism through which phytochemicals exert antinociceptive effects [34]. Limonene, a monoterpene commonly found in citrus fruits, acts as an antagonist at TRPV1 [35] and Transient Receptor Potential Ankyrin 1 (TRPA1) [36], reducing sensitivity to thermal and mechanical stimuli. In addition, limonene allosterically enhances the opening of Gamma-Aminobutyric Acid Type A receptor (GABA_A_) channels [37,38], increasing neuronal inhibition and helping to suppress allodynia and hyperalgesia in formalin and hot plate tests, with results comparable to some NSAIDs [39].

Regarding inflammation, phytochemicals focus on COX-2 inhibition and the attenuation of NF-κB signalling. These compounds hinder Inhibitor of kappa B alpha (IκBα) phosphorylation, preventing its degradation and the subsequent nuclear translocation of NF-κB, resulting in the decreased expression of pro-inflammatory cytokines such as TNF-α, IL-1β, and Interleukin-6 (IL-6) [40]. In primary microglial and astrocyte cultures, flavonoid-enriched extracts reduce the up-regulation of activation markers such as the cluster of differentiation molecule 11B (CD11b) and Ionized calcium-binding adapter molecule 1 (Iba-1), suggesting a protective effect against the chronic pro-inflammatory state typical of neuropathies [41].

The endocannabinoid system provides an additional axis of modulation: BCP acts as a selective CB_2_ agonist and inhibits the enzyme Monoacylglycerol lipase (MAGL), leading to increased 2-Arachidonoylglycerol (2-AG) levels and reduced inflammatory and neuropathic pain responses [42]. Complementary analyses have also shown that other terpenes, such as linalool and β-myrcene, modulate Adenosine A_2_A receptor (A_2_A) receptors and interact with serotonin transport, further expanding their analgesic potential [43,44].

### 2.2. Preclinical Animal Models of Pain

Selected terpenes, such as BCP, also engage nuclear receptors and kinase cascades. BCP has been shown to act as a CB_2_ receptor agonist that indirectly up-regulates Peroxisome Proliferator-Activated Receptor Gamma (PPAR-γ) and its co-activator Peroxisome Proliferator-Activated Receptor Gamma Coactivator 1-alpha (PGC-1α) in inflamed tissues, a mechanism supported by studies in arthritis and vascular inflammation models [45].

Several terpenoids modulate nociception through the direct or indirect engagement of opioid receptors, thereby enriching their multi-target analgesic profile. The neoclerodane diterpene salvinorin A, isolated from *Salvia divinorum*, is the first non-nitrogenous natural ligand identified as a high-affinity and highly selective κ-opioid receptor (KOR) agonist. The activation of KOR by salvinorin A produces potent antinociception in neuropathic and inflammatory pain models while sparing μ-opioid receptor (MOR)-mediated respiratory depression [46].

Among sesquiterpenes, BCP not only stimulates CB_2_ receptors but also provokes the peripheral release of the endogenous opioid β-endorphin; the resulting MOR-dependent signalling is demonstrated by the complete reversal of BCP-induced antinociception after pretreatment with naloxone or the MOR-selective antagonist β-funaltrexamine. Remarkably, sub-analgesic doses of BCP synergistically potentiate morphine, enabling a ≥ 40% dose reduction and attenuating tolerance development in capsaicin and formalin pain paradigms [47].

A comparable opioid-facilitating pattern is observed for the monoterpene linalool, whose inhibition of acetic acid-induced writhing and partial efficacy in hot plate assays are completely abolished by systemic naloxone, implicating the peripheral MOR activation and enhancement of endogenous opioid tone [48].

In addition to opioid-facilitating mechanisms, certain terpenes such as linalool also modulate glutamatergic neurotransmission by interacting with N-Methyl-D-Aspartate receptor (NMDA) receptors. Preclinical studies demonstrate that linalool acts as a non-competitive inhibitor of NMDA binding—directly reducing Tritium-labelled dizocilpine maleate binding in mouse cortical membranes—suggesting the inhibition of NMDA-mediated excitatory currents [49]. This mode of action contributes to its antinociceptive and anticonvulsant effects and may synergize with its opioid receptor engagement, offering the multimodal modulation of central sensitization and glutamate-induced neurotoxicity in pain pathways.

In addition to the mechanisms described above, terpenes attenuate neuro-immune amplification by directly repressing the Toll-like receptor-4 (TLR4) cascade, an innate immune hub now recognized as a pivotal driver of neuropathic pain chronification [50].

BCP lowers the TLR4 surface density on synovial macrophages and, in an ischemic brain, steers microglia toward an anti-inflammatory M2 phenotype, curbing TNF-α, IL-1β, and IL-6 release [51,52].

Central nervous system (CNS)-penetrant borneol interferes with hippocampal TLR4-NF-κB activation, restraining microglial M1 polarization and seizure-linked nociceptive sensitization [53].

Peripheral monoterpenes display similar behaviour: α-pinene dampens hepatic TLR4/NF-κB signalling in Carbon tetrachloride (CCl_4_)-induced fibrosis, and tea tree-derived α-terpineol inhibits TLR4-triggered IL-1β and IL-6 production in human macrophages [54,55].

Some monoterpenes, notably linalool and α-pinene, have been shown to act as positive allosteric modulators of GABA_A_ receptors. Electrophysiological studies reveal that linalool potentiates GABA-evoked currents at GABA_A_ receptors, enhancing inhibitory neurotransmission and contributing to its antinociceptive and anxiolytic action [56]. Likewise, α-pinene—and its metabolites myrtenol and verbenol—augment both synaptic and extrasynaptic GABA_A_ receptor activity, effects that are blocked by the benzodiazepine antagonist flumazenil, indicating action at the benzodiazepine-binding site [57].

Beyond their role as standalone treatments, the co-administration of phytochemicals with standard analgesics has revealed significant therapeutic synergies. For example, the combination of BCP oxide and paracetamol, tested in animal models, improves antinociceptive efficacy compared with either compound alone and mitigates the gastrointestinal side effects typical of NSAIDs, outlining a more favourable safety profile and opening avenues for combination drug approaches [58]. Preclinical studies have shown that specific cannabis-derived terpenes exert significant antinociceptive effects in rodent models of inflammatory, post-operative, and fibromyalgia-like pain [59]. Linalool, for example, reduced acetic acid-induced writhing in mice via peripheral opioidergic and cholinergic pathways, while terpene mixtures increased mechanical thresholds through adenosine A_2_A receptor activation [48]. These effects occurred without impairing motor function. Although these findings suggest a promising analgesic profile, particularly when compared with standard agents like morphine, they are limited to animal models and require confirmation in controlled human studies. These results justify the initiation of randomized controlled clinical trials to validate therapeutic protocols based on highly pure, high-concentration phytochemical extracts.

### 2.3. Overview of Clinical Studies

Although the majority of data on terpene-based analgesia derives from preclinical models, a number of small-scale clinical studies and observational reports suggest promising therapeutic effects in human pain conditions. These studies have investigated both isolated terpenes and complex phytochemical formulations containing terpene mixtures, often delivered via oral, transdermal, or inhalational routes.

In a musculoskeletal context, Farì et al. [60] conducted a 45-day interventional study in 38 patients with knee osteoarthritis. The subjects received oral supplementation with hemp seed oil enriched in BCP, myrcene, and ginger extract. Compared with an isocaloric terpene-free control, the intervention group showed significant reductions in pain and improvements in functional scores.

Ou et al. [61] assessed the effect of aromatherapy massages in 48 women suffering from primary dysmenorrhoea. The intervention cream contained 2.7% BCP, and the participants received abdominal massages daily during menstruation. The treatment significantly reduced both pain intensity and duration compared with the placebo (synthetic fragrance).

A randomized, double-blind, placebo-controlled crossover study by Wang et al. evaluated a THC-free oral formulation containing 300 mg CBD and micro-doses (1 mg each) of eight terpenes—including BCP—in 125 patients with chronic insomnia. The results showed an increase in the proportion of deep sleep (slow-wave sleep) by 1.3% on average, with maximal increases of up to 48 min per night in patients with low baseline values, and no adverse events were reported [62].

In another randomized placebo-controlled trial, a topical formulation containing 1% limonene was applied to the plantar fascia of patients with plantar fasciitis. Over 78% of subjects reported ≥85% pain reduction within ten days, a significantly greater effect than the placebo, which produced no substantial change [63].

A second clinical trial investigating limonene, in combination with menthol and gingerol, enrolled 56 patients with irritable bowel syndrome. After 30 days of oral supplementation (1.5% limonene), the patients showed a significant reduction in symptom scores, from “moderately ill” to “borderline,” with no reported adverse events and no major alterations in gut microbiota [64].

Finally, a crossover study in 20 healthy volunteers demonstrated that co-vaporizing limonene with THC selectively reduced the cannabinoid’s anxiogenic and paranoiagenic effects, without altering its pharmacokinetics or major psychoactive outcomes [65]. Although not strictly a pain study, these findings highlight the neuromodulatory capacity of terpenes in human subjects.

## 3. Cannabis Terpenes: Prevalence and Pharmacological Properties

Terpenes are volatile isoprenoid compounds found in many plants—including *Cannabis sativa* L.—and are responsible for their distinctive aromas. Recent scientific interest has focused on their possible role in cannabis’s medicinal properties, particularly their potential to modulate analgesia and contribute to the “entourage effect” alongside THC and CBD [66]. Over 100 terpenes have been identified in *Cannabis*, with profiles varying according to cultivar (chemovar), plant genetics, environmental conditions, and curing processes [67]. Generally, monoterpenes predominate in “sativa” varieties, while “indica” types are richer in earthy sesquiterpenes [68]. Terpene content in dried female inflorescences averages around 1% by weight, though highly aromatic chemovars can reach up to 3% [69].

### 3.1. Pharmacological Properties

Terpenes such as BCP (molecular weight, MW 204 Da), linalool (MW 154 Da), and α-pinene (MW 136 Da) fit comfortably inside Lipinski’s rule-of-five limits [70] for molecular weight and hydrogen bonding, yet most display log*p* values > 4, reflecting pronounced hydrophobicity and very low water solubility that restrict oral absorption and parenteral formulation [71]. In silico surveys confirm that many terpenes satisfy standard drug-likeness filters but trigger “poor solubility” or “high permeability” flags, forecasting erratic exposure after conventional dosing [72]. Their volatility further complicates manufacture and storage, while the absence of easily ionisable groups limits the opportunities for salt formation and pH-dependent solubility enhancement.

Unmodified BCP exhibits <10% absolute oral bioavailability in humans because of dissolution-limited absorption and extensive first-pass metabolism; a self-emulsifying drug delivery system (SEDDS) raised oral bioavailability, highlighting formulation as a critical determinant of systemic exposure [73]. Following inhalation, α-pinene shows rapid, perfusion-limited uptake with a pulmonary extraction ratio ≈ 0.6 and a total blood clearance of ≈1.1 h^−1^ kg^−1^, leading to short systemic residence times [74].

Phase I oxidation by cytochrome P450 enzymes predominates. Linalool undergoes allylic hydroxylation and epoxidation mainly via Cytochrome CYP2C19 and CYP2D6, generating polar metabolites eliminated in urine [75]. Limonene and its oxidized congener limonin potently inhibit Cytochrome CYP3A4 at low micromolar concentrations, posing a risk for food– or herb–drug interactions with narrow therapeutic index substrates [76]. Lavender oil terpenes similarly inhibit CYP3A4 and CYP1A2 activity in vitro, indicating possible metabolic competition with co-administered drugs [77].

Acute toxicity is generally low—myrcene has an oral LD_50_ > 5 g kg^−1^ in rodents, and dermal exposure is comparably non-lethal [78]. Nevertheless, chronic or high-dose exposure to certain monoterpenes (pulegone, menthofuran, and limonene) and sesquiterpenes (zederone and germacrone) has been linked to hepatocellular injury driven by reactive metabolites and oxidative stress [79]. Sensitization dermatitis and gastrointestinal irritation are additional, dose-related concerns [80]. Repeated high-dose BCP or linalool can prolong central nervous system depression in animal studies [81], but human evidence remains scarce and largely anecdotal.

Nano- and micro-emulsions, liposomes, and polymeric nanoparticles markedly enhance terpene solubility, protect against oxidative degradation, and extend circulation half-life; several preclinical studies report 2- to 10-fold gains in systemic exposure and sustained antinociception when terpenes are delivered in lipid-based carriers [82]. Alternative strategies involve chemical derivatization—either by introducing heteroatoms or by generating pro-drug conjugates—to lower lipophilicity, improve dissolution, and temper first-pass biotransformation [83]. Systemic exposure can also be increased by co-administering permeability enhancers such as piperine or cineole, or other metabolic inhibitors that blunt presystemic clearance [82,84,85], although the enzyme inhibition they elicit inevitably heightens the risk of clinically relevant herb–drug interactions.

### 3.2. Cannabis Terpenes Prevalence

The terpenes most abundantly represented in *Cannabis* are myrcene, limonene, pinene (α and β isomers), linalool, BCP, humulene, and terpinolene. Chemical analytical studies show that myrcene can constitute up to 40–65% of a cultivar’s total terpene content, typically corresponding to concentrations of about 0.3–0.8%, while BCP—also ubiquitous and abundant—is often present at 0.1–0.5% [86]. Other monoterpenes such as limonene, pinene, and linalool, and the sesquiterpene α-humulene, vary more widely according to the chemovar [87].

**Figure 1:**
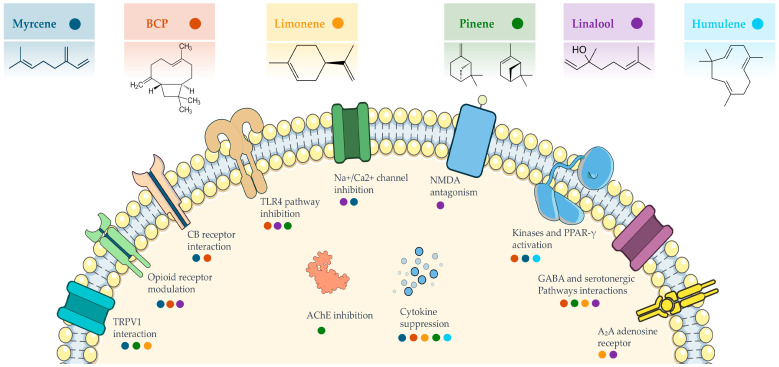
Illustration summarizing the main molecular targets and mechanisms of action of selected cannabis-derived terpenes involved in analgesic and anti-inflammatory pathways. Each coloured dot corresponds to a specific terpene and its associated interaction or effect on cellular signalling pathways. Abbreviations: BCP—β-Caryophyllene, TRPV1—Transient Receptor Potential Vanilloid 1, CB—Cannabinoid, TLR4—Toll-Like Receptor 4, Na^+^/Ca^2+^—Sodium/Calcium channels, NMDA—N-Methyl-D-Aspartate receptor, AChE—Acetylcholinesterase, PPAR-γ—Peroxisome Proliferator-Activated Receptor gamma, GABA—Gamma-Aminobutyric Acid, A_2_A—Adenosine receptor subtype A_2_A.

Several cannabis terpenes exhibit antinociceptive and anti-inflammatory activity in preclinical models through heterogeneous yet complementary mechanisms. BCP stands out for its “cannabinoid-like” action, selectively activating CB_2_ receptors. Monoterpenes such as myrcene, linalool, and limonene act on central and peripheral targets—including opioid, α_2_-adrenergic, and cholinergic receptors, TRP channels, and serotonergic pathways—thereby modulating pain perception and inflammation. In neuropathic animal models, certain cannabis-like terpene mixtures have demonstrated antinociceptive effects approaching those of morphine or synthetic cannabinoids [88]. These findings, however, are based on controlled preclinical settings and require cautious interpretation until confirmed in clinical trials. Figure 1 summarizes the principal mechanisms of action through which major cannabis-derived terpenes modulate nociception.

#### 3.2.1. Myrcene

Myrcene is an acyclic monoterpene (C_10_H_16_) and one of the most abundant terpenes in cannabis—especially in indica-dominant chemovars. It lends an earthy, musky aroma (it is also found in hops, lemongrass, and mangoes). In many modern cannabis varieties, myrcene accounts for >20% of the total terpene fraction. Typical dried flower levels range from roughly 0.1% to 0.5% *w/w*, although highly aromatic cultivars can reach ~1–2%; in extreme cases, myrcene has been reported to make up as much as ~65% of the total terpene profile.

The analgesic mechanisms of myrcene are not fully clarified, but several hypotheses have been proposed. A striking finding is that myrcene can act on TRP ion channels; Jansen et al. showed that micromolar concentrations of myrcene activated the TRPV1 (vanilloid) channel in rat cells, causing a Ca^2+^ influx—an effect blocked by the TRPV1 antagonist capsazepine [89]. However, a subsequent study by Heblinski et al. failed to reproduce myrcene-induced TRPV1 activation in human TRPV1-expressing cells [90], so the data on the TRP ion channels remain species- or context-specific.

Beyond TRP channels, myrcene appears to engage opioid pathways: in several rodent studies, naloxone partially reversed the antinociceptive effect of myrcene, implicating endogenous opioid receptors. Early work noted that myrcene’s analgesia resembles that of peripheral opioids (without central adverse events) and might be mediated by endogenous opioid release or the indirect modulation of opioid receptors [91].

Myrcene also exerts anti-inflammatory effects, relieving pain indirectly, by inhibiting inflammatory hyperalgesia driven by prostaglandin E_2_ and other mediators. While myrcene seems to act chiefly through non-cannabinoid routes [92], it has been shown to interact with local cannabinoid receptors (likely CB_2_) in murine models of joint inflammation, reducing pain and swelling, though without synergy with CBD [93]. Overall, myrcene’s analgesic action probably involves a combination of peripheral opioid receptor engagement, a reduction in inflammatory sensitization, and possibly TRPV1 modulation, although its precise molecular targets remain undefined.

Myrcene’s central nervous system effects are likely facilitated by its high lipophilicity and ability to readily cross the blood–brain barrier, enabling interaction with the neural substrates involved in nociceptive modulation [78]. These pharmacokinetic properties may underlie its reported sedative and antinociceptive actions observed in preclinical models.

Clarifying these mechanisms is particularly important given the prevalence of myrcene in cannabis chemovars and its potential to modulate multiple pain-related pathways. A deeper understanding could support its rational use in therapeutic formulations for chronic pain, where multifactorial modulation is often required.

Myrcene is popularly believed to produce sedative and anxiolytic action (the so-called “couch-lock” associated with high-myrcene cannabis [78]). Controlled studies are scarce, but one mouse study found that myrcene produced anxiolysis with marked sexual dimorphism [94]. The putative anxiolysis may stem from its sedative action or mild muscle relaxant effect. To date there are no direct human data on myrcene’s anxiolytic or antidepressant effects. Nevertheless, because chronic pain often co-exists with anxiety and poor sleep, the sedative–anxiolytic properties observed in animals could be clinically relevant for pain patients who are anxious and have difficulty sleeping.

#### 3.2.2. β-Caryophyllene

BCP is a bicyclic sesquiterpene (C_15_H_24_) with a spicy, peppery aroma, found in high concentrations in black pepper and cloves. In cannabis, BCP is often one of the dominant sesquiterpenes. It is detected in most sampled chemovars, although—compared with monoterpenes—it generally makes up a smaller share of the total terpene content [95]. Many medicinal cannabis varieties contain 0.1–0.5% BCP by weight, and in some (especially certain CBD-rich chemovars) it can be the primary terpene.

BCP engages numerous molecular targets and receptors, but it is best known as a selective agonist of the CB_2_ cannabinoid receptor [96]. BCP can also inhibit the TLR4 pathway and reduce the release of IL-1β, IL-6, and TNF-α from activated microglia [28,97]. These immunomodulatory actions contribute to analgesia by dampening inflammation and neuro-inflammation along pain pathways.

BCP exerts antinociceptive effects not only through CB_2_ receptor activation but also via the peripheral stimulation of endogenous β-endorphin release. This opioid-mediated mechanism is confirmed by the complete abolition of BCP-induced analgesia following pretreatment with naloxone or the MOR-selective antagonist β-funaltrexamine. Notably, BCP enhances the efficacy of morphine at sub-therapeutic doses, producing synergistic analgesia and enabling dose reductions in inflammatory pain models, while also mitigating the development of opioid tolerance [47].

Additional targets have been reported: BCP activates kinases (extracellular signal-regulated kinase, ERK 1/2; c-Jun N-terminal kinase, JNK) and the nuclear receptor PPAR-γ, which is involved in metabolic and anti-inflammatory signalling [98]. BCP-induced analgesia is likewise sensitive to opioid antagonists in some peripheral pain assays, suggesting crosstalk with the opioid system. In one study, locally injected BCP attenuated capsaicin-evoked pain through combined CB_2_ and peripheral opioid mechanisms [99].

Preclinical evidence for BCP as an analgesic and anti-inflammatory agent is robust, particularly in chronic pain models. Rodent studies consistently show that systemic or local BCP reduces pain behaviours in diverse paradigms: inflammatory pain (formalin test, acetic acid writhing), neuropathic pain (nerve injury or chemotherapy-induced), and visceral pain [28]. Prophylactic BCP even prevented neuropathic pain development in an antiviral-induced model [100].

Although BCP exhibits robust antinociceptive and anti-inflammatory activity through selective CB_2_ receptor agonism, its translational development is hindered by the absence of standardized dosing protocols and limited pharmacodynamic characterization in humans. Rigorous clinical investigations are needed to establish optimal dosing, therapeutic windows, and inter-individual variability in efficacy.

Beyond pain and inflammation, BCP has shown benefits in models of anxiety, spasms, seizures, depression, alcohol dependence, and Alzheimer’s disease [101]. In an experimental study on mice, BCP produced anxiolytic, antidepressant, and anticonvulsant activity by modulating benzodiazepine/GABA_A_ receptors, serotonergic pathways, and the L-arginine/nitric oxide pathway; its anxiolysis was abolished by flumazenil and bicuculline, while the co-administration of L-arginine blunted all three effects, confirming a mechanism dependent on reduced nitric oxide synthesis [102].

These data broaden BCP’s pharmacological profile, suggesting clinical potential as a multisystem modulator of anxiety disorders, mood disorders, and epilepsy.

Human data, though still limited, are encouraging. In the musculoskeletal field, Farì et al. reported that 45 days of supplementation with hemp seed oil enriched in BCP, myrcene, and ginger extract significantly reduced pain and improved functional scores in 38 patients with knee osteoarthritis versus an isocaloric terpene-free control [60]. In gynecology, Ou et al. showed that daily abdominal massage with an aromatherapy cream containing 2.7% BCP shortened the duration and intensity of primary dysmenorrhoea in 48 women, outperforming a synthetic fragrance placebo [61]. In a placebo-controlled crossover study of 125 insomnia patients, Wang et al. found that a THC-free oral formulation composed of 300 mg CBD plus micro-doses (1 mg each) of eight terpenes, including BCP, increased deep sleep proportion by an average of 1.3%, with gains of up to 48 min per night in participants with low baseline values and no adverse effects [62]. These preliminary studies—coupled with BCP’s favourable safety profile—suggest that adding BCP to phytotherapeutic formulations may be a promising strategy for improving sleep quality and alleviating various forms of pain and anxiety, warranting larger-scale clinical trials.

#### 3.2.3. Limonene

Limonene is a cyclic monoterpene (C_10_H_16_) with a strong citrus aroma, abundantly present in the peels of citrus fruits. In cannabis it is a common monoterpene, although its levels vary widely by cultivar. In a survey of North American cannabis, limonene ranked among the eight most prevalent terpenes and is often dominant in sativa-type chemovars [103]. The typical limonene content in dried flowers is 0.1–0.3%, but it can exceed 0.5% in lemon-scented varieties [104].

Limonene is known for its centrally mediated anxiolytic and anti-stress properties, yet its direct antinociceptive targets are less well characterized. It binds neither cannabinoid nor opioid receptors with a high affinity. Its analgesic actions appear to derive from it anti-inflammatory and neuromodulatory effects. Limonene has been shown to lower pro-inflammatory cytokines such as TNF-α and IL-1β in animal models [105]. A 2024 preclinical study found that d-limonene dramatically reduced post-operative peritoneal adhesion formation in rats by suppressing Transforming Growth Factor Beta 1 (TGF-β1), TNF-α, and Vascular Endothelial Growth Factor (VEGF) and restoring antioxidant enzymes, indicating marked anti-fibrotic and anti-angiogenic activity [106].

Araújo-Filho et al. (2020) demonstrated in a mouse sciatic nerve injury model that 28 days of daily limonene administration accelerated nerve regeneration, lessened mechanical hyperalgesia, and reduced spinal astrogliosis; these effects were linked to the suppression of IL-1β/TNF-α and increased Growth-Associated Protein 43 (GAP-43), NGF, and phosphorylated extracellular signal-regulated kinase (p-ERK) in the spinal cord [107]. Limonene also modulates the adenosinergic system: a recent study showed that its anxiolytic effect in mice is mediated by the A_2_A adenosine receptors [37]; this is notable because this receptor’s signalling also influences pain processing [88].

Clinically, a double-blind, randomized, placebo-controlled trial found that a topical terpene formulation containing 1% limonene as a skin permeation enhancer produced an ≥85% pain reduction in over 78% of plantar fasciitis patients within ten days, whereas the placebo yielded no significant improvement [63]. Additional data reinforce this profile: a double-blind, placebo-controlled trial in 56 patients with irritable bowel syndrome showed that 30 days of supplementation with menthol, limonene, and gingerol (1.5% limonene) lowered the symptom score from “moderately ill” to “borderline” with no adverse events and no significant microbiota changes, suggesting that low oral doses of limonene can enhance irritable bowel syndrome therapy [64]. Neuro-behaviourally, a crossover study in 20 healthy volunteers found that co-vaporizing limonene with THC selectively blunted the cannabinoid’s anxiogenic and paranoid effects, while leaving other pharmacodynamic and pharmacokinetic parameters unchanged [65].

Taken together, the evidence indicates that limonene confers analgesic efficacy chiefly in inflammatory and neuropathic pain models—likely by damping cytokine-driven hyperalgesia—while it is ineffective for acute nociceptive pain and can be an irritant at high local doses. Its greatest clinical promise may lie in adjuvant use, improving mood and the immune milieu in chronic pain patients rather than serving as a stand-alone analgesic.

#### 3.2.4. Pinene

Pinene is a bicyclic monoterpene (C_10_H_16_) with the characteristic resinous scent of pine, found in pine resin, rosemary, and many other evergreen conifers. Cannabis often contains both α-pinene and β-pinene isomers. Pinene is common across cannabis chemovars—a survey detected α-pinene in nearly every sample, albeit usually as a minor constituent (often <0.1–0.5%) [108]. Certain cultivars—particularly some “sativa” types or hybrids—reach higher pinene levels and display a distinct pine aroma (e.g., Jack Herer, which can contain ~1% total pinene). Pinene has attracted interest for its potential cognitive benefits: it may counteract THC-induced memory deficits by inhibiting acetylcholinesterase [66].

Pinene is a bronchodilator; both α- and β-isomers may improve airflow to the lungs [109]. These compounds can cross the blood–brain barrier and, at high doses, exhibit mild sedative or anxiolytic effects, possibly through GABA_A_ modulation [110].

The analgesic mechanisms of pinene are not fully elucidated. The incomplete knowledge of its mechanism of action is partly due to the limited number of studies employing pure pinene; many utilize essential oils rich in pinene [57].

α-pinene has demonstrated significant anti-inflammatory properties in various preclinical models [111]. It is believed to inhibit the NF-κB pathway and the production of pro-inflammatory cytokines such as TNF-α and IL-6. According to the study by Fakhri et al., α-pinene may produce antinociceptive outcomes via interaction with the opioid system, as this response was partially inhibited by naloxone [112]. Additionally, a possible modulation of TRPV1 channels has been suggested, although evidence is less direct compared with other terpenes [113].

β-pinene, although less studied than its α-isomer for analgesia, shares anti-inflammatory properties and has been shown to inhibit prostaglandin E_2_ (PGE_2_) production [114].

Studies on animal models of inflammatory pain have shown that essential oils containing pinene as a major component can reduce edema and leukocyte migration [115]. The anti-inflammatory activity of pinene has also been associated with its ability to reduce oxidative stress [116]. Despite its promising preclinical activities, robust clinical studies on the analgesic efficacy of isolated pinene in humans are lacking. Its role in the “entourage effect” is often cited, particularly for its ability to mitigate some adverse effects of THC, such as short-term memory impairment, due to its inhibitory activity on acetylcholinesterase [117]. The inhibition of acetylcholinesterase can enhance cholinergic neurotransmission, but its contribution to analgesia is uncertain [118].

#### 3.2.5. Linalool

Linalool is a linear monoterpenic alcohol (C_10_H_18_O) with a delicate floral fragrance. It is a minor terpene in most cannabis strains, typically present at 0.01–0.5%. Only some specialized strains (often indica-dominant) feature linalool as the dominant terpene; in most chemovars, it contributes only marginally to the terpene profile [108]. Despite its low concentrations, linalool’s aroma is easily perceptible and pharmacologically potent. Linalool is notably found in lavender and is a key component of lavender essential oil, which has been used for centuries as a calming agent [119,120].

Linalool acts on various neurochemical targets, and for this reason it is among the terpenes with the broadest spectrum of action. It has been shown to modulate glutamatergic transmission by non-competitively inhibiting the dizocilpine maleate (MK-801) site of the NMDA receptor and competitively reducing glutamate binding, without interfering with direct GABAergic activity [121]. This action contributes to the reduction in neuronal hyperexcitability associated with central sensitization. Additionally, it inhibits specific voltage-dependent sodium and calcium channels, further limiting nociceptive transmission [122]. This means that linalool can reduce excitatory neurotransmission, which is crucial in the central sensitization of pain. Batista et al. demonstrated that the antinociceptive effect of linalool in a glutamate-induced pain model was due to interference with ionotropic glutamate receptors (NMDA, AMPA, and kainate) [123].

Some studies suggest the involvement of adenosine A_1_/A_2_A receptors, likely through an increase in extracellular levels induced by the inhibition of nitric oxide production and the activation of the nitric oxide–cyclic Guanosine Monophosphate pathway (NO–cGMP) [43]. At high concentrations, it may act as a GABA_A_ antagonist in vitro, but in vivo it shows an enhancement of inhibitory tone, probably through indirect mechanisms [56,124].

Similar to BCP, linalool exerts an opioid-facilitating effect shown by its analgesic action in the acetic acid writhing test, and to a lesser degree in the hot plate assay. Moreover, it is fully reversed by naloxone, indicating the involvement of peripheral MOR activation and endogenous opioid release [48].

Additionally, in models of cisplatin-induced hyperalgesia, linalool down-regulates the renal expression of TLR4, attenuating oxidative stress and pain via the suppression of pro-inflammatory signalling cascades [125].

Centrally, linalool exerts sedative, anxiolytic, and hypnotic effects, with modulation also mediated by the olfactory pathway [126]. Inhalation in rodents induces analgesia and sedation dependent on the integrity of the olfactory bulb and the hypothalamic orexinergic system, suggesting an interaction between sensory inputs and limbic circuits [127]. This supports the hypothesis of an olfactory–limbic mechanism justifying the efficacy of aromatherapy in certain pain syndromes.

Numerous animal models support the antinociceptive efficacy of linalool. In acute thermal nociception, Peana et al. demonstrated a significant increase in pain threshold in mice with systemic doses of 50–100 mg/kg, comparable or superior to those of morphine [43]. It has been proved that linalool may even reduce behavioural responses to irritating chemical stimuli (acetic acid, capsaicin, and glutamate) [128].

In models of chronic inflammatory hyperalgesia and neuropathic pain (e.g., spinal nerve ligation), linalool has shown robust analgesic effects, with a significant reduction in mechanical allodynia [129].

Linalool exhibits marked anxiolytic activity, which is potentially useful in clinical contexts where pain is accompanied by psycho-emotional distress. Clinical studies on lavender oil, containing about 30% linalool, have documented anxiety reduction comparable to lorazepam [130]. These results are particularly relevant for conditions like fibromyalgia, where anxiety and insomnia co-exist with chronic pain. Proposed mechanisms include the modulation of the dopaminergic D_2_ and muscarinic M_2_ receptors, as well as the involvement of the orexinergic system [57].

Controlled studies on patients with knee osteoarthritis demonstrated significant pain reductions after massages with lavender oil [131]. This effect has also been reported for dysmenorrhea and post-herpetic pain [61,132]. Although large-scale randomized controlled clinical trials are lacking, preliminary evidence justifies further investigation into its use in chronic pain treatment, particularly in disorders with high affective or central components.

#### 3.2.6. Humulene

Humulene, or α-caryophyllene, is a monocyclic sesquiterpene with the molecular formula C_15_H_24_, a structural isomer of the better-known BCP. Its presence is documented in various aromatic and medicinal plants, including hops, sage, and ginseng, where it contributes to characteristic aromatic profiles, such as the hoppy note of beer [133]. In *Cannabis sativa* chemovars, α-humulene is frequently co-expressed with BCP, sharing part of its biosynthesis, and its concentration in dried flowers generally ranges between 0.01% and 0.3%. Some varieties with a high BCP content also show a significant co-expression of humulene, contributing to a more complex terpene profile with woody and earthy nuances [108].

From a pharmacological standpoint, α-humulene is less characterized than its isomer, especially regarding its role in pain mechanisms. Its anti-inflammatory activity is well documented, particularly in essential oil preparations containing this sesquiterpene [134]. Proposed mechanisms include the inhibition of the NF-κB-mediated inflammatory cascade and a reduction in pro-inflammatory cytokines such as IL-1β, despite the absence of direct interaction with the CB_1_ and CB_2_ cannabinoid receptors [135]. A pharmacologically relevant action, distinct from that of BCP, involves the inhibition of the COX-2 enzyme, as demonstrated by Fernandes et al., who observed reduced PGE_2_ levels in animal models treated with humulene-containing extracts [134]. These findings suggest an activity like that of NSAIDs but mediated by a terpene with a profoundly different chemical structure.

Although systematic studies on isolated α-humulene in pain management are not available, numerous works on oils and plant extracts containing it provide indirect data on its analgesic potential. The essential oil extracted from *Peperomia serpens*, containing approximately 11.5% humulene, has demonstrated efficacy in reducing pain and inflammation in murine models, with effects only partially antagonized by naloxone, suggesting the involvement of non-opioid analgesic pathways [136]. Studies conducted on extracts from *Cordia verbenacea* and *Pterodon pubescens*, rich in humulene and BCP, have reported significant antinociceptive and anti-inflammatory activity, likely synergistic, with reductions in edema and hyperalgesia in models of acute inflammation, primarily through the inhibition of prostaglandin synthesis [137,138]. Furthermore, hop extracts with substantial humulene concentrations (approximately 15%) have been associated with reduced adjuvant-induced arthritis in rats, along with the decreased expression of TNF-α and COX-2, further supporting the compound’s anti-inflammatory role [139]. The topical use of humulene in analgesic creams—although currently supported more by anecdotal evidence than by robust clinical data—suggests potential utility in the treatment of localized pain, particularly when formulated in combination with other anti-inflammatory compounds such as BCP [30].

Centrally, humulene is not commonly associated with anxiolytic or sedative effects, in contrast to other terpenes such as linalool or myrcene. Its action appears to be primarily focused on the peripheral inflammatory component of pain.

From a clinical standpoint, the documentation on α-humulene remains extremely limited. No published clinical studies currently explore its analgesic or anti-inflammatory efficacy in human subjects. Therefore, future investigations should aim to isolate α-humulene and evaluate its effects in well-designed human trials, particularly in chronic pain syndromes. Such studies would help clarify its individual therapeutic potential and determine whether it offers clinically meaningful advantages either as a stand-alone agent or in synergistic phytocomplexes.

To provide a structured synthesis of the evidence discussed in this review, Table 1 presents a comprehensive overview of the principal preclinical studies investigating the analgesic potential of the cannabis-derived terpenes discussed in this review, including the key experimental outcomes, proposed mechanisms of action, and putative therapeutic applications.

In parallel, Table 2 summarizes the results of clinical investigations, detailing the effects observed in human subjects, molecular targets, and potential indications.

### 3.3. Acute Versus Chronic Analgesic Profiles of Cannabis Terpenes

Preclinical data show that BCP suppresses nocifensive behaviour in acute inflammatory assays such as the formalin first and second phases, and it continues to alleviate allodynia during multi-day treatment in neuropathic models like partial sciatic ligation, with significant analgesia persisting beyond two weeks of oral administration [99,140]. Linalool affords rapid antinociception in acetic acid writhing and hot plate tests—effects abolished by naloxone—and, when given repeatedly, attenuates cisplatin-induced mechanical hyperalgesia over a 10-day period via the down-regulation of TLR4 signalling [48,125]. Myrcene reduces writhing and prolongs hot plate latency within minutes of acute dosing; moreover, multi-week topical or oral delivery lessens pain and inflammation in rat adjuvant mono-arthritis without loss of potency [91,93]. Limonene mirrors this pattern, producing naloxone-sensitive antinociception in writhing and formalin assays, while 28-day daily administration reverses mechanical allodynia and spinal cytokine up-regulation in sciatic nerve ligation neuropathy [107,141]. Finally, pinene acutely elevates hot plate latency and lowers writhing counts, and chronic inhalation or oral treatment prevents mechanical hypersensitivity in partial sciatic ligation neuropathy with an efficacy comparable to gabapentin [57].

These findings collectively support the notion that the terpenes reviewed herein exhibit clinically relevant analgesic properties, particularly in the context of chronic pain. Their multimodal mechanisms of action and favourable safety profiles justify further investigation as potential stand-alone therapeutics or as opioid-sparing adjuvants in the long-term management of persistent pain syndromes.

### 3.4. Safety Profile of Cannabis Terpenes

From a safety perspective, the terpenes discussed in this review generally exhibit a favourable toxicological profile. Most of these compounds are naturally present in foods or herbs that have been consumed by humans for centuries, and many have been granted GRAS (Generally Recognized As Safe) status by the U.S. Food and Drug Administration (FDA) [142]. For instance, limonene is a widely used aromatic food additive that, even when administered at high doses (up to 8 g/day) in clinical trials involving oncology patients, has only elicited mild adverse effects, primarily transient gastrointestinal discomfort [28].

Linalool, derived from lavender, has been used for decades in aromatherapy and cosmetic formulations; adverse reactions are rare and typically limited to allergic contact dermatitis in predisposed individuals—oxidized linalool can act as a hapten in certain cases [80]. Similarly, BCP, a constituent of culinary spices, displays excellent tolerability. Preclinical studies report a wide safety margin, with very high oral LD_50_ values and no evidence of psychotropic or opioid-like effects [143].

Nevertheless, it is important to acknowledge that high doses of pure terpenes, particularly when administered via non-traditional routes such as injection, can elicit adverse events. For example, injectable monoterpenes at supraphysiological doses have induced central nervous system depression in rodents, characterized by deep sedation, ataxia, hypothermia, and occasional local tissue irritation [81]. Such outcomes are predictable given their lipophilic nature; at elevated concentrations, these compounds may disrupt neuronal membranes in a manner reminiscent of general anesthetics. However, the doses required to elicit such effects far exceed those relevant to any foreseeable therapeutic use in humans.

In the context of inhalational applications, the principal concern pertains to airway irritation. Highly volatile terpenes such as menthol, especially at elevated concentrations, have been documented to provoke airway irritation, bronchoconstriction, chest tightness, and dyspnoea, particularly in sensitive individuals and children, potentially through the activation of TRPA1 receptors or histamine release [144]. Similarly, cineole, while beneficial at therapeutic doses, can irritate mucosa when administered in undiluted form, and is contraindicated in certain asthmatics due to its potential to exacerbate respiratory symptoms [145]. Accordingly, aromatherapy practices employ diluted formulations (typically < 0.5–2%) and controlled exposure durations to minimize such risks.

Regarding pharmacokinetic interactions, certain terpenes have been shown to modulate hepatic drug-metabolizing enzymes. Limonene and pinene, for instance, have demonstrated a moderate inhibition of specific cytochrome P450 isoforms (such as CYP2C9 and CYP2D6) in vitro, whereas β-caryophyllene and nerolidol may influence P-glycoprotein activity and drug transport mechanisms [146]. These interactions could theoretically alter the metabolism of co-administered medications (e.g., increasing the plasma levels of drugs that are CYP substrates) if terpenes are present at sufficiently high concentrations. However, in standard herbal preparations, the terpene content is generally too low to produce clinically relevant interactions. Nonetheless, this possibility warrants caution in the context of highly concentrated supplements or intensive aromatherapy, particularly in polypharmaceutical patients.

A final consideration involves the challenge of blinding in clinical studies. The strong and distinctive odour of many terpenes complicates the design of truly double-blind, placebo-controlled trials, as both patients and investigators may recognize the scent and thereby distinguish active treatments from placebo. This introduces a potential source of bias in aromatherapy research and necessitates creative methodological approaches—for example, using control oils with distinctly different but equally pleasant and potentially calming aromas to separate specific pharmacological effects from nonspecific psychological responses to olfactory stimuli.

In conclusion, cannabis-derived terpenes appear to be safe and well tolerated at the therapeutic dosages supported by current evidence. No significant adverse events have emerged in small-scale human trials, aside from occasional reports of mild sedation, hypotension, or cutaneous reactions in sensitive individuals.

### 3.5. Limitations and Remedial Strategies

Despite their promising pharmacological activities, terpenes face several limitations that hinder their clinical development. These include poor water solubility, high volatility, low oral bioavailability, and extensive first-pass metabolism, which collectively result in unpredictable pharmacokinetics and limited systemic exposure after conventional dosing [71,73,75]. Additionally, their hydrophobic nature and the absence of ionisable groups restrict formulation options, particularly for parenteral or aqueous delivery [72].

Toxicological concerns also emerge at supraphysiological concentrations, especially when terpenes are administered as isolated compounds or through non-conventional routes. Certain monoterpenes have demonstrated central nervous system depression, hepatocellular injury, or airway irritation in preclinical models, effects rarely observed in traditional use but relevant when considering pharmaceutical applications at high doses [79,80,81,144,145].

To overcome these challenges, several technological and pharmacological remedies have been proposed. Nanoformulations, such as liposomes, micro- and nano-emulsions, and polymeric nanoparticles, have been shown to enhance solubility, protect against oxidative degradation, and prolong systemic circulation, resulting in improved bioavailability and sustained pharmacological action [82]. Alternatively, SEDDS and pro-drug strategies have demonstrated potential to improve absorption and reduce first-pass metabolism [61,71,73,83].

Another viable approach involves the co-administration of absorption enhancers (e.g., piperine or cineole) or enzyme inhibitors, although these must be used cautiously due to the risk of herb–drug interactions [84,85]. Finally, advances in synthetic chemistry and molecular docking are enabling the design of semi-synthetic derivatives or terpene analogues with improved physicochemical and pharmacokinetic properties.

Collectively, these strategies may help unlock the full therapeutic potential of cannabis-derived terpenes by addressing key limitations related to delivery, metabolism, and safety.

### 3.6. Comparative Advantage over Conventional Analgesics

Although certain terpenes can elicit toxic or allergic reactions—particularly at supratherapeutic doses or when applied in concentrated forms—their overall safety profile remains favourable, especially when compared with conventional pain medications. For instance, NSAIDs are associated with a well-documented risk of gastrointestinal bleeding, renal toxicity, and cardiovascular events, especially with chronic use [18]. Opioid analgesics, despite their efficacy, carry serious risks of tolerance, dependence, respiratory depression, and opioid-induced hyperalgesia [20]. Even anticonvulsants and antidepressants used in neuropathic pain often produce dose-limiting central side effects such as sedation, dizziness, or cognitive impairment [19].

In contrast, many terpenes act on multiple pain-related targets, including cannabinoid, opioid, GABAergic, and TRP receptor systems, as well as inflammatory pathways such as COX-2 and NF-κB. This multi-target profile may enable the effective modulation of both nociceptive and neuropathic components of pain without relying on single-receptor mechanisms that often lead to tolerance or side effects. Moreover, selected terpenes like β-caryophyllene and linalool have demonstrated the capacity to enhance the efficacy of opioids or NSAIDs at lower doses, reducing the need for higher—and more toxic—doses of standard drugs [47,58].

From a regulatory perspective, several terpenes are classified as GRAS for human consumption, with decades of empirical use in foods, cosmetics, and aromatherapy [142]. While hypersensitivity reactions can occur, especially in predisposed individuals, the incidence remains low and generally manageable with appropriate formulation strategies and dose control.

Therefore, when used in well designed, titratable preparations and within pharmacologically justified dose ranges, cannabis-derived terpenes may offer safer, more sustainable, and potentially synergistic alternatives or complements to traditional analgesics—particularly in the context of chronic, multifactorial pain.

## 4. Discussion

This review highlights the therapeutic potential of cannabis-derived terpenes in pain management, while also pointing out significant gaps in the scientific literature—particularly concerning the translation of preclinical findings to the clinical context. The overwhelming majority of evidence arises from animal models or in vitro studies, with a marked scarcity of rigorously conducted randomized controlled trials in humans. A major concern is the discrepancy between the doses used in preclinical research and those realistically achievable in humans. Indeed, doses administered to rodents (50–100 mg/kg) are substantially higher than those attainable through cannabis flower consumption (≤20 mg total), potentially leading to an overestimation of antinociceptive effects. Moreover, the pharmacokinetic profile of terpenes is characterized by low bioavailability, high lipophilicity, rapid hepatic metabolism, and a short half-life, complicating the identification of effective concentrations at target sites. The lack of systematic and comparative pharmacokinetic studies further limits the development of a reliable therapeutic rationale.

The analgesic properties reported for individual terpenes are highly dependent on the experimental model employed. For example, limonene exhibits efficacy in inflammatory pain models [126], whereas BCP appears more effective against mechanical allodynia. However, the absence of meta-analyses and the prevalence of studies reporting positive outcomes raise concerns about publication bias. The limited number of direct comparisons with conventional analgesics (NSAIDs, opioids) restricts the comparative evaluation of efficacy, although some estimates suggest that myrcene may reach up to 60% of morphine’s effect and that BCP may exhibit potency comparable to acetylsalicylic acid [134]. Nonetheless, these estimates lack independent validation. Furthermore, preclinical results are subject to significant interspecies variability and methodological heterogeneity, which complicate extrapolation to human contexts. This underscores the urgent need for integrated pharmacokinetic and pharmacodynamic studies in humans. Despite these limitations, the mechanistic diversity and favourable safety profiles of many terpenes continue to support their promise as novel modulators in pain therapy.

The so-called “entourage effect”—whereby terpenes are thought to synergize with phytocannabinoids to enhance analgesic outcomes—remains insufficiently supported by experimental data. In vitro studies have failed to demonstrate the direct modulation of CB_1_ or CB2 receptors by terpenes, and clinical trials suggest that the antinociceptive activity of cannabis is primarily attributable to THC. Nevertheless, certain combinations of terpenes with each other or with cannabinoids such as CBD have shown additive or synergistic effects on alternative targets, including inflammatory pathways, oxidative stress, and non-cannabinoid neurotransmitter systems [30]. A more comprehensive evaluation would require the inclusion of affective, functional, and biomolecular endpoints to capture the nuances of such interactions.

Another issue concerns the generalization of data obtained using synthetic terpenes or extracts from non-cannabis plant sources, which may contain co-active constituents or impurities absent from cannabis phytocomplexes. Cannabis terpene profiles are highly heterogeneous and shaped by complex interactions among multiple bioactive molecules. Preclinical studies, however, tend to evaluate isolated terpenes under conditions that do not reflect real-world exposure. Furthermore, terpene interactions with metabolic pathways may significantly affect the pharmacodynamics and pharmacokinetics of co-administered phytocompounds.

Many studies focus exclusively on reflexive responses to mechanical or thermal stimuli [129], overlooking core aspects of chronic pain, including spontaneous pain, affective and cognitive components, and functional impact. The use of advanced animal models incorporating affective–motivational behavioural scales could yield a more realistic assessment of therapeutic efficacy [30]. Additionally, the possibility that observed antinociceptive effects are due to sedation or motor impairment rather than true analgesia necessitates the systematic application of motor control assays and pain biomarkers.

The increasing commercial interest in the production and promotion of cannabis-based preparations exposes the scientific literature to potential conflicts of interest. Ensuring rigorous transparency in research funding and author disclosures is essential to prevent the enthusiasm surrounding the entourage effect or the “natural” use of terpenes from overshadowing an objective appraisal of the available evidence.

In summary, while biological data support a potential analgesic role for terpenes, the current body of clinical evidence is insufficient to warrant their systematic integration into therapeutic practice. Future research must advance toward robust experimental protocols, active comparators, and the in-depth exploration of specific mechanisms of action for each terpene. Only through well-designed, randomized clinical trials will it be possible to definitively establish the role of cannabis terpenes in the treatment of chronic pain.

## 5. Conclusions

In conclusion, a thorough analysis of the current literature reveals that, despite their structural and functional heterogeneity, cannabis-derived terpenes represent a promising frontier in analgesic research. Compounds such as BCP, myrcene, limonene, linalool, and α-humulene have demonstrated significant antinociceptive activity in preclinical models, mediated by multiple mechanisms of action often distinct from those of classical cannabinoids. These findings highlight the therapeutic potential of cannabis-derived terpenes as multimodal modulators of nociceptive signalling, yet their clinical translation remains hindered by limited human data and the need for pharmacokinetic standardization. Rather than broadly modulating inflammatory pathways, individual terpenes exert defined molecular actions: BCP activates CB_2_ receptors and down-regulates NF-κB signalling; limonene suppresses TNF-α expression and attenuates microglial activation; linalool reduces IL-1β and modulates glutamatergic transmission; and α-humulene inhibits COX-2 and PGE_2_ production. Future research should prioritize well-designed clinical trials to validate these mechanistic insights, establish safe and effective dosing regimens, and explore terpene synergy with cannabinoids in personalized analgesic strategies. However, the enthusiasm generated by the pharmacological activity observed in animal models must be tempered by an awareness of the numerous methodological limitations that still affect the field. The lack of controlled clinical trials, the scarcity of terpene-specific pharmacokinetic data, the use of non-translatable dosing regimens, and the absence of direct comparisons with standard analgesics all restrict the generalizability of current findings. Moreover, while the “entourage effect” remains an appealing hypothesis, it requires rigorous validation through multifactorial experimental designs and the identification of clinically meaningful endpoints that extend beyond basic nociceptive behavioural metrics.

It is also important to acknowledge the substantial interspecies differences that affect the extrapolation of preclinical data to human contexts. Variability in receptor distribution, signalling pathway sensitivity, metabolic enzyme expression, and blood–brain barrier permeability can markedly alter both the pharmacokinetics and pharmacodynamics of bioactive terpenes across species. These discrepancies may lead to the over- or under-estimation of efficacy and safety profiles when translating animal model outcomes to human applications. Therefore, while rodent models offer valuable mechanistic insights, their predictive validity for human analgesic responses remains inherently limited, reinforcing the necessity for well-designed translational and clinical studies.

Looking ahead, the path toward the genuine clinical applicability of analgesic terpenes will necessarily depend on randomized trials, the development of optimized bioavailability formulations, pharmacological interaction studies with cannabinoids and conventional drugs, and a refinement of preclinical models to incorporate affective and functional dimensions of pain. Only through an integrated, methodologically robust, and clinically oriented approach will it be possible to determine whether terpenes can evolve from promising bioactive molecules into validated therapeutic agents for the management of chronic pain.

## 6. Future Perspectives

In the coming decade, it will be essential to systematically translate preclinical evidence on terpenes into clinical applications through the development and implementation of advanced-phase randomized clinical trials. These studies should employ standardized terpene formulations delivered via optimized bioavailability systems—such as pressurized inhalers or titratable oral matrices. Such trials must integrate pharmacokinetic and pharmacodynamic analyses, including validated pain assessment scales, the quantification of inflammatory and neurochemical biomarkers, and evaluations of functional outcomes.

Concurrently, it will be critical to initiate mechanistic investigations at both the molecular and systemic levels, leveraging state-of-the-art technologies such as CRISPR/Cas9 gene editing, integrated omics analyses (transcriptomics and proteomics), and advanced functional neuroimaging. These tools will enable the precise mapping of terpene targets—including non-canonical pathways—and inform the rational design of novel terpene analogues with optimized pharmacological profiles in terms of efficacy, selectivity, and metabolic stability.

Moreover, terpenes must be incorporated into personalized and multimodal therapeutic strategies, which are tailored to the phenotypic profile of pain and the patient’s pharmacogenetic background. The objective is to maximize clinical benefit, reduce adverse events, and solidify the foundation of an innovative therapeutic paradigm—one grounded in replicable evidence and methodologically rigorous research—for the management of chronic pain.

## Figures and Tables

**Table 1:** Summary of preclinical studies evaluating the analgesic potential of selected cannabis-derived terpenes.

Terpenes (Class)	Chemotype Prevalence	Experimental Outcomes	Mechanisms	Potential Uses	Refs
BCP (S)	CBD-rich strains	Antinociception in formalin, writhing, neuropathy, arthritis; allodynia prevention; synergy with morphine/paracetamol	CB_2_ agonist, β-endorphin release; ↓MAGL, ↓COX-2, ↓IL-6, ↓TLR4, ↑PPAR-γ, ↓NF-κB; microglia M2 polarization, ERK/JNK modulation	Multi-target analgesic, opioid-sparing	[28,42,45,47,51,52,58,98,99,100,140]
Myrcene (M)	Indica-dominant	↓Writhing, ↓edema/arthritis, ↑hot plate latency; sedative–anxiolytic in mice	TRPV1 modulation, peripheral opioid pathways, COX-2 inhibition, local CB_2_, Nav and Cav interaction; ↓p38 MAPK, ↓glutamate release, ↓PGE_2_	Inflammatory/arthritic pain, sedative adjunct	[78,89,90,91,93,94]
Limonene (M)	Sativa-type	Sciatic nerve protection, antioxidant; ↓allodynia, ↓cytokines, ↓peritoneal adhesions	NF-κB/TNF-α/IL-1β inhibition; A_2_A-dependent; TRPA1 modulation, GABA_A_ modulation, NO–cGMP pathway modulation	Neuropathy, post-surgical adhesions, visceral inflammation	[36,37,105,106,107,141]
Pinene (M)	Common to all chemovars	↓Formalin and hot plate pain, ↓edema; improved neuropathic hyperalgesia	↓NF-κB, ↓PGE_2_; GABA_A_ modulation, AChE inhibition, possible TRPV1 and opioid pathways	Anti-inflammatory analgesic, cognitive support	[109,110,111,112,113,115,116]
Linalool (M)	Indica-dominant	Antinociception (writhing, hot plate, glutamate); ↓neuropathic allodynia, ↓cisplatin hyperalgesia	NMDA and voltage-gated Na^+^/Ca^2+^ blockade; ↑A_1_/A_2_A, ↓TRPA1, opioid, and ↓TLR4, ↓microglial activation/↓IL-6, ↓AMPA/kainate, GABA_A_ modulation	Inflammatory/neuropathic pain, anxiolytic	[43,48,57,59,121,122,123,124,125,127,128,129]
Humulene (S)	With BCP-rich strains	↓Edema, ↓hyperalgesia, anti-arthritic, gastroprotective	COX-2 and NF-κB inhibition, cytokine suppression; ↓IL-1β, ↓TNF-α, ERK/JNK modulation	Topical anti-inflammatories, arthritis	[134,135,136,137,138,139]

Abbreviations: ↑: Disinhibition; ↓: Inhibition; BCP = β-Caryophyllene; S = Sesquiterpene; CBD = Cannabidiol; M = Monoterpene; CB_2_ = Cannabinoid receptor type 2; TRPV1 = Transient receptor potential vanilloid 1; TRPA1 = Transient receptor potential ankyrin 1; TLR4 = Toll-like receptor 4; PPAR-γ = Peroxisome proliferator-activated receptor gamma; NF-κB = Nuclear factor kappa-light-chain-enhancer of activated B cells; COX-2 = Cyclooxygenase-2; PGE_2_ = Prostaglandin E_2_; NMDA = N-methyl-D-aspartate receptor; GABA_A_ = Gamma-aminobutyric acid type A receptor; A_2_A = Adenosine A_2_A receptor; MAGL = Monoacylglycerol lipase; ERK = Extracellular signal-regulated kinase; JNK = c-Jun N-terminal kinase.

**Table 2:** Overview of clinical studies evaluating the effects of cannabis terpenes in pain-related and comorbid conditions.

Terpenes	Clinical Effects	Mechanisms	Potential Uses	Refs
BCP	↓pain and ↑function in knee osteoarthritis (*n* = 38); ↓intensity/duration of dysmenorrhoea (*n* = 48); ↑deep sleep in primary insomnia (*n* = 125)	Presumed CB_2_ activation ± β-endorphin release (not measured)	Osteoarthritis, dysmenorrhoea, sleep disorders comorbid with pain	[60,61,62]
Myrcene	↓pain, ↑ function vs. control (*n* = 38)	Hypothesized peripheral opioid-like action (not clinically tested)	Nutraceutical support in osteoarthritis	[60]
Limonene	≥85% pain reduction in plantar fasciitis (*n* = 62); ↓IBS symptoms (*n* = 56); attenuation of THC-induced anxiety/paranoia (*n* = 20 HV)	Topical anti-inflammatory; A_2_A modulation; cutaneous TRPA1 involvement	Musculoskeletal pain, IBS, psycho-emotional modulator in cannabis therapy	[63,64,65]
Linalool	↓pain in knee OA after lavender oil massage (*n* = 90); ↓post-herpetic pain (*n* = 524); benefit reported in dysmenorrhoea (*n* = 48)	GABA potentiation; NMDA modulation; peripheral opioid pathways	Aromatherapy for chronic musculoskeletal pain, cutaneous neuropathic disorders	[61,131,132]
Pinene	rapid uptake and clearance (*n* = 8)	–	Candidate for future trials (bronchodilation, memory)	[74]
Humulene	no clinical pain studies available	–	Topical anti-inflammatory candidate	–

Abbreviations: BCP = β-Caryophyllene; CB_2_ = Cannabinoid receptor type 2, THC = Δ9-tetrahydrocannabinol; IBS = Irritable bowel syndrome; HV = Health Volunteers; OA: Osteoarthritis; TRPA1 = Transient receptor potential ankyrin 1; A_2_A = Adenosine A_2_A receptor, NMDA = N-methyl-D-aspartate receptor; GABA—Gamma-Aminobutyric Acid; ↑: Disinhibition; ↓: Inhibition.

## Data Availability

No new data were created or analyzed in this study. Data sharing is not applicable to this article.

## Abbreviations

The following abbreviations are used in this manuscript:

BCP	β-caryophyllene
CB_2_	Cannabinoid receptor type 2
TRP	Transient receptor potential
NF-κB	Nuclear Factor kappa-light-chain-enhancer of activated B cells
COX-2	Cyclooxygenase-2
IL-1β	Interleukin 1 beta
TNF-α	Tumour Necrosis Factor alpha
ACC	Anterior cingulate cortex
BNST	Bed nucleus of the stria terminalis
NSAIDs	Nonsteroidal anti-inflammatory drugs
Nav	Voltage-gated sodium channel
Cav	Voltage-gated calcium channel
Kv	Voltage-gated potassium channel
TRPV1	Transient receptor potential vanilloid 1
THC	Δ9-Tetrahydrocannabinol
CBD	Cannabidiol
CB_1_	Cannabinoid receptor type 1
RCTs	Randomized controlled trials
MAPK	Mitogen-activated protein kinase
PKC	Protein Kinase C
TRPA1	Transient Receptor Potential Ankyrin 1
GABA_A_	Gamma-Aminobutyric Acid Type A receptor
IκBα	Inhibitor of kappa B alpha
IL-6	Interleukin-6
CD11b	Cluster of differentiation molecule 11B
Iba-1	Ionized calcium-binding adapter molecule 1
MAGL	Monoacylglycerol lipase
2-AG	2-Arachidonoylglycerol
A_2_A	Adenosine A_2_A receptor
PPAR-γ	Peroxisome Proliferator-Activated Receptor Gamma
PGC-1α	Peroxisome Proliferator-Activated Receptor Gamma Coactivator 1-alpha
KOR	κ-opioid receptor
MOR	μ-opioid receptor
NMDA	N-Methyl-D-Aspartate receptor
TLR-4	Toll-like receptor-4
CCl_4_-	Carbon tetrachloride
SEDDS	Self-emulsifying drug delivery system
ERK	Extracellular signal-regulated kinase
JNK	c-Jun N-terminal kinase
TGF-β1	Transforming Growth Factor Beta 1
GAP-43	Growth-Associated Protein 43
NGF	Nerve Growth Factor
PGE_2_	Prostaglandin E_2_
MK-801	Dizocilpine maleate
NO–cGMP	Nitric Oxide–cyclic Guanosine Monophosphate pathway
GRAS	Generally Recognized As Safe
FDA	Food and Drug Administration
